# Differential Gene Expression at Coral Settlement and Metamorphosis - A Subtractive Hybridization Study

**DOI:** 10.1371/journal.pone.0026411

**Published:** 2011-10-31

**Authors:** David C. Hayward, Suzannah Hetherington, Carolyn A. Behm, Lauretta C. Grasso, Sylvain Forêt, David J. Miller, Eldon E. Ball

**Affiliations:** 1 Evolution, Ecology and Genetics, Research School of Biology, Australian National University, Canberra, Australian Capital Territory, Australia; 2 Biomedical Science and Biochemistry, Research School of Biology, Australian National University, Canberra, Australian Capital Territory, Australia; 3 ARC Centre of Excellence for Coral Reef Studies and School of Pharmacy and Molecular Sciences, James Cook University, Townsville, Queensland, Australia; Oregon State University, United States of America

## Abstract

**Background:**

A successful metamorphosis from a planktonic larva to a settled polyp, which under favorable conditions will establish a future colony, is critical for the survival of corals. However, in contrast to the situation in other animals, e.g., frogs and insects, little is known about the molecular basis of coral metamorphosis. We have begun to redress this situation with previous microarray studies, but there is still a great deal to learn. In the present paper we have utilized a different technology, subtractive hybridization, to characterize genes differentially expressed across this developmental transition and to compare the success of this method to microarray.

**Methodology/Principal Findings:**

Suppressive subtractive hybridization (SSH) was used to identify two pools of transcripts from the coral, *Acropora millepora*. One is enriched for transcripts expressed at higher levels at the pre-settlement stage, and the other for transcripts expressed at higher levels at the post-settlement stage. Virtual northern blots were used to demonstrate the efficacy of the subtractive hybridization technique. Both pools contain transcripts coding for proteins in various functional classes but transcriptional regulatory proteins were represented more frequently in the post-settlement pool. Approximately 18% of the transcripts showed no significant similarity to any other sequence on the public databases. Transcripts of particular interest were further characterized by *in situ* hybridization, which showed that many are regulated spatially as well as temporally. Notably, many transcripts exhibit axially restricted expression patterns that correlate with the pool from which they were isolated. Several transcripts are expressed in patterns consistent with a role in calcification.

**Conclusions:**

We have characterized over 200 transcripts that are differentially expressed between the planula larva and post-settlement polyp of the coral, *Acropora millepora*. Sequence, putative function, and in some cases temporal and spatial expression are reported.

## Introduction

**Figure 1:**
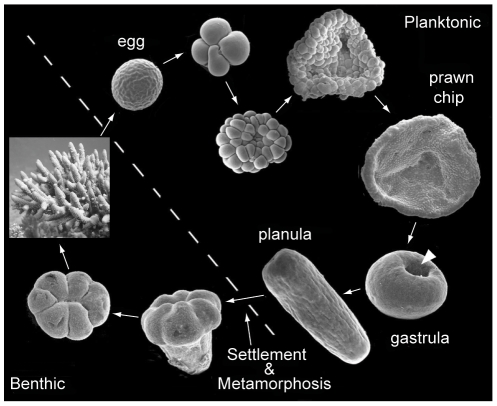
The life cycle of the coral, *Acropora millepora*. The *Acropora* life cycle begins with a mass spawning event, when all of the corals in a given area spawn simultaneously. Buoyant egg-sperm bundles are released which break up on their way to the surface, releasing the sperm to fertilize eggs from other colonies. Once fertilized, the egg soon begins to divide, reaching the prawn chip stage in about 12 hours. Then, in a process which is not fully understood, the prawn chip rounds up to form a gastrula. As the blastopore (white arrowhead) closes, cilia develop and the spherical embryo begins to rotate. Over the next several days the sphere gradually elongates to form a spindle-shaped planula larva and the nervous system and nematocysts develop. After a period of a few days to a few months in the plankton, where it swims aboral end first, the planula begins a searching behavior of corkscrew swimming into the bottom. When it finds appropriate chemical settlement cues it rounds up and metamorphoses into a primary polyp. During this process the aboral end of the planula is resorbed and the oral end expands, resulting in a post-settlement, crown-shaped form from which the primary polyp arises. This polyp grows and produces others, eventually resulting in a colony with thousands of polyps.

Corals have a biphasic life cycle with planktonic juvenile stages and benthic adults (figure 1). These two phases are separated by settlement and metamorphosis, which are critical stages in coral development, as they mark the transition from a swimming larval stage to a sedentary polyp, which will found a new colony [1], [2], [3] In *Acropora* and other corals this transition involves a number of processes, among the most important being receipt of settlement cues, profound changes in morphology, building of new tissues, commencement of calcification, and uptake of symbionts. These processes are ultimately under genetic control, involving expression changes in a large number of genes many of which have been characterized in previous microarray studies [4]. Other studies on *Acropora* have focused on specific genes or gene families (e.g. the Pax genes [5], the integrins [6] [7] and the Sox genes [8]. There is also a rapidly growing comparative literature on developmental processes in other corals (e.g. [9] [10] and a large literature on the role of genes in development of the sea anemone *Nematostella vectensis* (see [11] for a recent summary).

High throughput sequencing has led to a revolution in the amount of sequence data available or obtainable relatively cheaply. As a result of using this technology the full genome sequence of the coral *Acropora digitifera* has recently been published [12] and the genome sequence of *Acropora millepora* is available (http://coralbase.org/). In addition, extensive transcriptome databases are becoming available for different developmental stages of *A millepora* [13] , Weiss et al in prep). High throughput sequencing is also a powerful technique for quantifying changes in gene expression before and after a developmental event or application of a stressor.

Another, older way of isolating differentially expressed genes is by using suppressive subtractive hybridization (SSH), a technique which enables the isolation of sequences which are more abundant in one mRNA population compared to another. This technique has been used to identify coral genes which are differentially expressed between aposymbiotic and symbiotic polyps [14], during thermal stress [15], and during exposure to high solar irradiance [16] as well as to study ascidian metamorphosis [17].

Here, we have used SSH to focus on changes occurring at settlement and metamorphosis by comparing mRNA isolated from pre-settlement planula larvae to that isolated from newly settled polyps. The resulting set of differentially expressed transcripts complements and extends those already identified by microarray [4]. Because the powerful genetic tools that allow direct functional analysis in model invertebrates such as *Drosophila* and *Caenorhabditis* have yet to be developed for corals, there are presently three principal ways by which we can attempt to infer the function of a specific gene. Firstly, we can compare gene expression before and after the start of a specific process. For example, calcification starts at settlement, facilitating the isolation of genes involved in this process. Secondly, we can infer function from the sequence of a gene and comparison with the activity of similar genes in other systems. Finally, we can examine the temporal and spatial distribution of the transcript, which can help us to evaluate the likelihood of the putative function. We have utilized all of these approaches in the present study in an attempt to characterize the likely function of genes differentially expressed before and after settlement.

## Results

### Suppressive subtractive hydridization

Suppressive subtractive hybridization (Clontech PCR-Select) was used to compare gene expression changes in settlement-competent planula larvae, and post-metamorphosis primary polyps. This procedure produced as its end result two pools of plasmid clones containing short cDNA inserts; one enriched for sequences corresponding to transcripts present at higher levels in larvae and the other, conversely, enriched for sequences more highly expressed in the primary polyp. We refer to the former of these pools as the “A” pool and the latter as the “B” pool; clones and sequences derived from each pool are named with the appropriate letter.

In total 234 clones from pool A and 246 clones from pool B were sequenced. These were found to represent 137 unique sequences from pool A and 138 unique sequences from pool B due to the fact that some sequences were independently isolated more than once (Supporting Information Figure S1). No overlap was found between the pools. The sequences were compared to the available coral EST and transcriptome databases [13], [18], [19], [20] (Weiss et al, unpublished) using blastn. In the majority of cases a single sequence could be assigned to a single transcript. However, there were nine cases in which two or more pool A sequences correspond to different regions of the same transcript (Supporting Information Table S1). This is not unexpected since the SSH procedure results in the isolation of RsaI restriction fragments which have an average size of approximately 250 bp. In three cases, a single pool A clone sequence was comprised of two RsaI fragments which correspond to different transcripts. This is presumably due to two separate fragments being ligated together during the cloning procedure. Similarly, in the case of pool B, thirteen transcripts correspond to more than one pool B sequence, and there were six examples of pool B clones containing more than one RsaI fragment, corresponding to different transcripts (Supporting Information Table S2). One of the pool A sequences was found to correspond to a set of closely related transcripts, one of which was found in pool B. While these may represent different genes, the level of nucleic acid similarity is high enough to allow cross hybridization; these sequences were removed from the dataset. One of the pool B transcripts contains a highly repetitive sequence, possibly artefactual, and was discarded. This resulted in a total of 116 transcripts corresponding to pool A and 121 transcripts corresponding to pool B. The predicted proteins coded for by the transcripts, and the nucleic acid sequences and accession numbers are presented in Supporting Information Tables S1 and S2.

### Functional categories

The sequences were sorted into functional categories using the results of blast and protein domain searches (Tables 1 and 2, Supporting Information Tables S1 and S2). Gene Ontology annotations are shown in Supporting Information Tables S3 and S4. Of the pool A transcripts, 79 could be annotated, 20 had database hits but no functional annotation and 17 had no significant database hit. A similar distribution was observed for the pool B transcripts where 84 sequences could be annotated, 11 had database hits with no functional annotation and 26 had no significant similarity to the databases. Annotated transcripts are shown in Tables 1 and 2; lists of all transcripts, including description of blast hits are given in Supporting Information Tables S1 and S2.

**Table 1:** Pool A transcripts annotation.

transcript	class	name
A63	calcium homeostasis	calretinin
A25	cell replication/cell division	Rad17
A43	cytoskeleton proteins	dynein, cytoplasmic, intermediate polypeptide 2
A44	cytoskeleton proteins	Actin
A54	cytoskeleton proteins	Myosin light polypeptide 6
A56	cytoskeleton proteins	Actin
A67	cytoskeleton proteins	actin-related protein 2
A87	cytoskeleton proteins	Tctex-1
A111	cytoskeleton proteins	echinoderm microtubule-associated protein-like 1
A2	extracellular matrix (ECM)/cell adhesion proteins	ZP-EGF protein
A9	extracellular matrix (ECM)/cell adhesion proteins	vW TSP domain protein
A12	extracellular matrix (ECM)/cell adhesion proteins	Trefoil
A26	extracellular matrix (ECM)/cell adhesion proteins	Collagen
A32	extracellular matrix (ECM)/cell adhesion proteins	EGF-like domain protein
A52	extracellular matrix (ECM)/cell adhesion proteins	vWA domain protein
A69	extracellular matrix (ECM)/cell adhesion proteins	zonadhesin
A90	extracellular matrix (ECM)/cell adhesion proteins	cnidarian egg lectin isoform d
A93	extracellular matrix (ECM)/cell adhesion proteins	Trefoil
A102	extracellular matrix (ECM)/cell adhesion proteins	vW TSP domain protein
A39	heat shock/response to cell damage	AlkB
A74	heat shock/response to cell damage	FKBP-type peptidyl-prolyl cis-trans isomerase
A1	immunity	MACPF apextrin
A27	immunity	TIR
A35	immunity	pentraxin
A16	intracellular signal transduction	SH2 domain protein
A33	intracellular signal transduction	RasGAP
A77	intracellular signal transduction	guanine nucleotide-binding protein
A84	intracellular signal transduction	Bruton's tyrosine kinase-like protein
A13	ion-binding/ion transport	Calcium channel
A17	ion-binding/ion transport	AN1-type zinc finger and ubiquitin domain-containing protein
A5	metabolism	N-terminal nucleophile aminohydrolase
A6	metabolism	Pyridoxal-dependent decarboxylase
A7	metabolism	Lipase
A23	metabolism	Phophatidylserine decarboxylase
A57	metabolism	short chain dehydrogenase
A60	metabolism	glycinamide ribonucleotide synthetase
A66	metabolism	sepiapterin reductase
A89	metabolism	Very long chain acyl-CoA dehydrogenase
A94	metabolism	oxidoreductase
A15	oxidative stress	thyroxine 5′-deiodinase
A41	oxidative stress	DnaJ
A55	oxidative stress	Ferritin
A30	proliferation/growth/development	leucine zipper-EF-hand
A61	proliferation/growth/development	calmodulin
A14	proliferation/growth/development	ADP-ribosylation factor
A11	protease	Astacin family protease
A34	protease	Peptidase
A36	protease	Calpain
A53	protease	ADAM metallopeptidase with thrombospondin type 1 motif
A71	protease	serine protease
A104	protease	Astacin family protease
A20	protein synthesis (translation)	Ribosomal protein L32
A31	protein synthesis (translation)	Queuine tRNA-ribosyltransferas
A95	protein synthesis (translation)	ribosomal protein L15
A109	protein synthesis (translation)	Eukaryotic translation initiation factor 4E binding protein
A92	regulation	fidgetin-like
A64	RNA-binding proteins, RNA processing	U2-associated SR140 protein
A65	RNA-binding proteins, RNA processing	poly A binding protein
A101	RNA-binding proteins, RNA processing	mRNA cap guanine-N7 methyltransferase
A50	signalling	LWamide
A80	signalling	CRY1
A19	transcription-related proteins	THAP domain protein
A37	transcription-related proteins	zinc finger protein
A108	transcription-related proteins	lysine-specific histone demethylase 1A
A116	transcription-related proteins	zinc finger protein 16-like
A3	unknown	red fluorescent protein
A4	unknown	A.millepora C012-D9
A8	unknown	A.millepora GS01TE02
A40	unknown	SCP domain protein
A42	unknown	WD40 domain protein
A58	unknown	14-3-3
A70	unknown	WD repeat domain 48-like
A72	unknown	14-3-3
A78	unknown	hypothetical protein [*Acropora tenuis*]
A79	unknown	WD40 domain protein
A96	unknown	dpy-19
A97	unknown	ArgGlu rich1
A99	unknown	heme binding protein 2
A100	unknown	early estrogen-induced gene 1

Functional annotation of pool A transcripts. Pool A transcripts are sorted into functional classes based on the results of blast and protein domain searches. The columns contain the following information: transcript, the transcript number as referred to in the text; class, the functional class to which the transcript belongs; name, the name we have assigned to the transcript.

**Table 2:** Pool B transcripts annotation.

transcript	class	name
B42	calcium homeostasis	calreticulin
B9	cell replication/cell division	RNA polymerase
B71	cell replication/cell division	proliferating cell nuclear antigen
B75	cell replication/cell division	protein phosphatase 6 regulatory subunit 3
B36	cytoskeleton proteins	Tubulin-specific chaperone C
B57	cytoskeleton proteins	coactosin-like
B64	cytoskeleton proteins	FERM myosin regulatory light chain interacting protein
B113	cytoskeleton proteins	twinfilin
B5	extracellular matrix (ECM)/cell adhesion proteins	DOMON domain
B12	extracellular matrix (ECM)/cell adhesion proteins	DOMON domain
B17	extracellular matrix (ECM)/cell adhesion proteins	laminin gamma
B26	extracellular matrix (ECM)/cell adhesion proteins	ZP domain mesoglein
B84	extracellular matrix (ECM)/cell adhesion proteins	FRED domain
B85	extracellular matrix (ECM)/cell adhesion proteins	Galaxin-like2
B93	extracellular matrix (ECM)/cell adhesion proteins	DOMON domain
B4	immunity	CEL-III Lectin
B49	immunity	C-type lectin
B21	intracellular signal transduction	guanine nucleotide-binding protein subunit beta-2-like 1
B32	intracellular signal transduction	Ras protein
B48	intracellular signal transduction	protein tyrosine phosphatase
B55	intracellular signal transduction	GPCR
B97	intracellular signal transduction	SH3
B99	intracellular signal transduction	SH3 and PX domains 2A
B112	intracellular signal transduction, transport	Ran
B14	ion-binding/ion transport	HC03- transporter
B43	ion-binding/ion transport	transporter
B44	ion-binding/ion transport	transient receptor potential cation channel
B6	metabolism	Asp/Glu racemase
B24	metabolism	carbonic anhydrase
B33	metabolism	5-aminolevulinate synthase-like
B40	metabolism	Glycosyl hydrolase 31
B47	metabolism	mitochondrial malic enzyme 3
B60	metabolism	aldo-keto reductase
B66	metabolism	isocitrate lyase
B73	metabolism	carbonic anhydrase
B81	metabolism	Hydrolase
B88	metabolism	beta galactosidase
B89	metabolism	S-adenosylhomocysteine hydrolase-like 2
B101	metabolism	epidermal retinol dehydrogenase 2
B102	metabolism	lipase member K
B104	metabolism	zinc-binding alcohol dehydrogenase
B123	metabolism	adenosine monophosphate deaminase 2
B23	oxidative stress	peroxiredoxin 4-like
B74	oxidative stress	protein disulfide isomerase
B46	proliferation/growth/development membrane traffciking	target of myb1-like
B19	protease	peptidase
B59	protease	mitochondrial-processing peptidase subunit beta
B100	protease	protease
B116	protease	proteasome subunit beta type-2
B62	protease, apoptosis	cathepsin-L-like
B10	proteases, degradation	proteasome 26S subunit
B27	proteases, degradation	astacin-like
B69	protein synthesis (translation)	isoleucine tRNA synthetase
B2	response to stress	small cysteine-rich protein 3
B18	response to stress	SCRIP small cysteine-rich protein 6
B122	response to stress	Universal stress protein
B103	RNA-binding proteins	cold shock domain-containing protein E1, RNA binding
B111	signalling	fibroblast growth factor
B16	transcription-related proteins	NK-4 homeobox protein
B20	transcription-related proteins	PaxD
B34	transcription-related proteins	myeloid leukemia factor 1-interacting protein
B37	transcription-related proteins	zinc finger protein GLIS3
B52	transcription-related proteins	ERG
B53	transcription-related proteins	nuclear factor erythroid 2-related factor 3
B63	transcription-related proteins	Pirin
B77	transcription-related proteins	B-cell translocation gene
B78	transcription-related proteins	GATA zinc finger domain-containing
B79	transcription-related proteins	Pax-3
B80	transcription-related proteins	modulator of activity of Ets
B82	transcription-related proteins	FoxO
B87	transcription-related proteins	forkhead domain
B92	transcription-related proteins	GA-binding protein alpha chain
B105	transcription-related proteins	H1 histone family
B30	transport proteins	SUMO
B95	transport proteins	Betaine/Carnitine/Choline transporter
B3	unknown	hypothetical protein A047-G9 [Acropora millepora]
B15	unknown	transmembrane receptor
B25	unknown	TPR repeat protein 24
B61	unknown	TPR_2
B70	unknown	small glutamine-rich TPR-containing protein
B91	unknown	ubiquitin domain-containing protein
B107	unknown	SCP domain
B109	unknown	14-3-3 epsilon
B110	unknown	BSD domian

Functional annotation of pool B transcripts. Pool B transcripts are sorted into functional classes based on the results of blast and protein domain searches. Column headings are as for Table 1.

Both pools contain transcripts coding for proteins for general cell growth and maintenance functions such as cell division and replication, cytoskeleton, metabolism and protein synthesis. While this category may be thought of as consisting of housekeeping genes, it includes examples of genes that may be expected to be expressed at different levels in different cells, or during development. For example, many metabolic enzymes may exhibit regulated expression during development. In addition, differences in the rates of cell division between the planula and polyp could result in some of the housekeeping transcripts being expressed at different relative levels and, as a result, appearing in the screen.

Both pools contain examples of transcripts coding for extracellular matrix/cell adhesion proteins. However the types of protein in the two pools differ. In pool A there are four extracellular matrix proteins containing vWA domains (three with thrombospondin domains) and two trefoil domain proteins which, in other animals, are associated with tissues containing mucus-producing cells [21]. In pool B there are three examples of membrane-bound proteins containing DOMON domains, which may bind heme or sugar ligands [22]. Both pools contain transcripts coding for proteins with signaling functions as well as several proteases and stress response proteins.

Pool A has four transcripts coding for transcriptional regulatory proteins. Pool B contains fourteen transcripts coding for proteins in this category, including two Pax proteins, two forkhead domain proteins and two Ets domain proteins.

### Temporal expression levels

**Figure 2:**
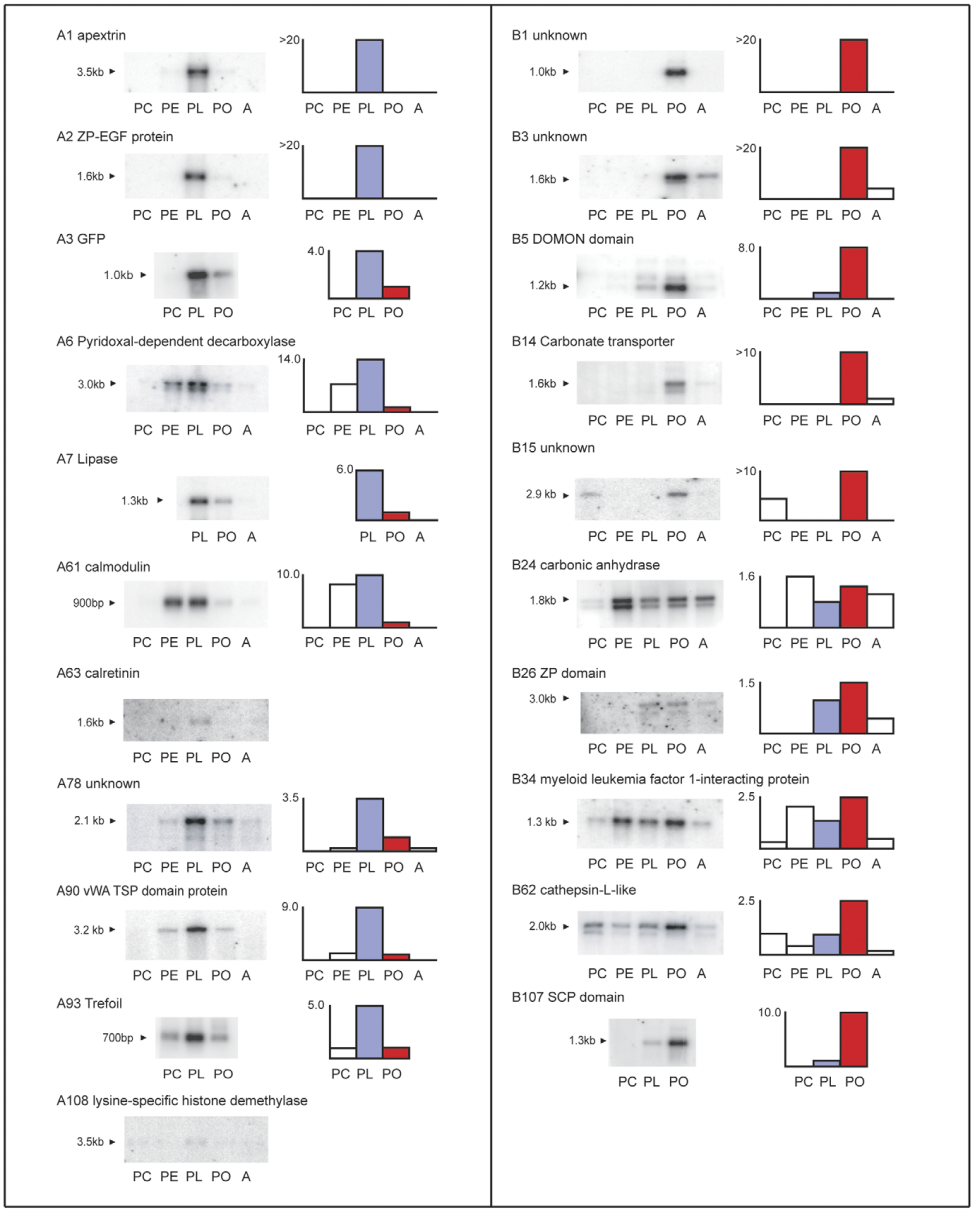
Virtual northern blots. Virtual northern blots provide more detailed information as to the expression of transcripts identified by SSH. This figure is organized so that genes up-regulated pre-settlement are in the left column, arranged in the order of their occurrence in Table 1. Genes up-regulated post-settlement are in the right column, arranged in the order of their occurrence in Table 2. The virtual northern blots are shown on the left with the stages from which RNA was made, designated PC (for prawn chip, a pre-gastrulation stage), PE (for pear, a stage after the blastopore has closed and the spherical embryo has begun to elongate), PL (for pre-settlement planula, the elongate larval stage which may be of extended duration), PO (for the immediate post-settlement stage) and A (for the adult colony). The sizes of the detected transcripts are indicated beside the blots. Accompanying each blot is a quantitative diagram of signal intensity at the various stages, with the pre-settlement (PL) intensity shown in purple and the post-settlement (PO) intensity shown in red. Numbers on the ordinate indicate the ratio of PL to PO intensities for the pool A blots and of PO to PL intensities for the pool B blots.

The expression of several of the sequences was measured using virtual northern blots to confirm whether there was differential expression between the planula and polyp stages and to get a fuller description of the changes in expression during development. Virtual northern blots are useful where material is scarce, and use cDNA amplified in a limited cycle PCR in the place of mRNA [23]. Such blots allow relative levels of expression and the size of the transcript to be measured. The results are shown in Figure 2.

The virtual northern blots indicate that the SSH process was largely successful in isolating differentially expressed sequences. In all cases, sequences from pool A are expressed at a higher level in the planula than in the primary polyp; conversely, pool B sequences have higher levels of expression in the polyp. Some of the sequences are particularly highly expressed, as indicated by the strength of the signal on the virtual northern blot, for example pool A transcripts A1 and A2, and pool B transcripts B1 and B3. These sequences were used to screen cDNA libraries, and this abundance was reflected in the high number of plaques obtained (not shown). They were also isolated multiple times from the pools of PCR-Select colonies. This is probably a reflection of their very high abundance, so that in spite of the normalization which occurs during the SSH procedure they remain highly represented. Other sequences, represented only once in the SSH pools, have varying levels of expression, but even when the level of expression is low, for example in the cases of pool A transcripts A63 and A108, there is still evidence of differential expression.

Most of the sequences show a dynamic developmental pattern, with a pronounced peak of expression at the stage from which the sequence was isolated. In several cases the ratio of expression levels between the planula and primary polyp is ten-fold or greater. For the pool A transcripts this includes an innate immunity gene, apextrin (A1, [24]), and sequences coding for an extracellular matrix protein containing EGF and Zona Pellucida domains (A2), a decarboxylase (A6), and a calmodulin (A61). Highly differentially regulated pool B transcripts include B14 coding for a HC03- transporter, and four transcripts of unknown function, B1, B3, B15 and B107. Interestingly, one of these, B15, has similarity to a cDNA sequence isolated during a screen for transcripts differentially expressed during ascidian metamorphosis [17]. Another pool B transcript encoding a DOMON domain protein (B5) is also highly up-regulated in the primary polyp. Other pool A sequences showing high levels of differential regulation include transcripts coding for a larval fluorescent protein (A3, [25] [26]), an extracellular matrix protein (A90), a trefoil factor protein (A93), and a lipase (A7). Transcript A78 is similar to an *A. tenuis* transcript isolated in a screen for genes that show an increase in expression in symbiotic juvenile polyps compared to those lacking zooxanthellae [14]. The high degree of up-regulation of these transcripts in the relevant developmental stage is strongly suggestive of a functional role.

### Spatial Expression patterns

**Figure 3:**
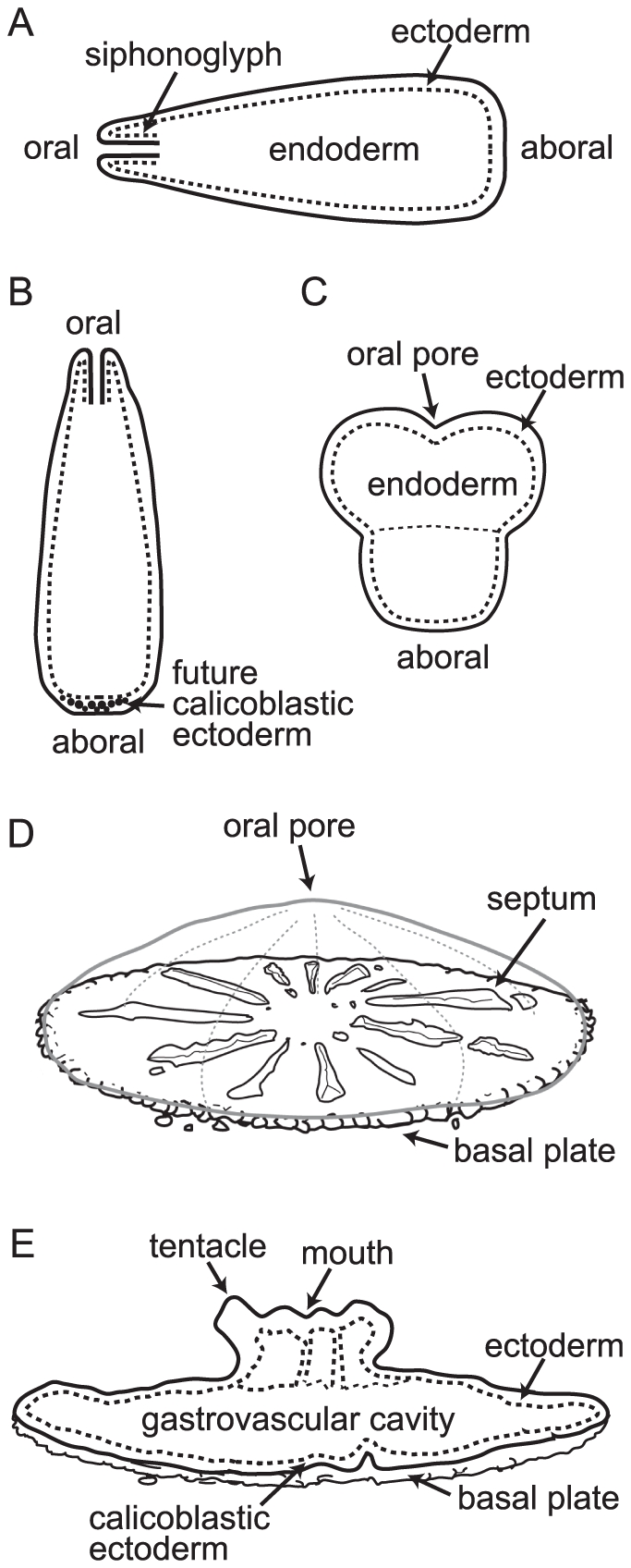
Schematic diagram labeling the morphological features discussed in the text and figure captions. (A) A planula larva swimming horizontally pre-settlement has a lipid filled endoderm surrounded by a ciliated ectoderm. The siphonoglyph is an infolding of the body wall at the oral pore. Swimming direction is aboral end first. (B) When ready to settle, the planula begins searching the bottom until it encounters appropriate chemical cues. The majority of planulae then round up and settle. However, morphology is variable and specimens such as that shown in (C) are often found; these may represent larvae which have started to settle and then rejoined the plankton. (D) The planula settles on its aboral end and calcification starts in the space between the calicoblastic ectoderm and the substratum. Once a calcified plate has been laid down vertical skeletal elements (septa) start to be formed, dividing the plate into segments. (E) Six tentacles develop around the mouth of the first polyp as its column elongates.

**Figure 4:**
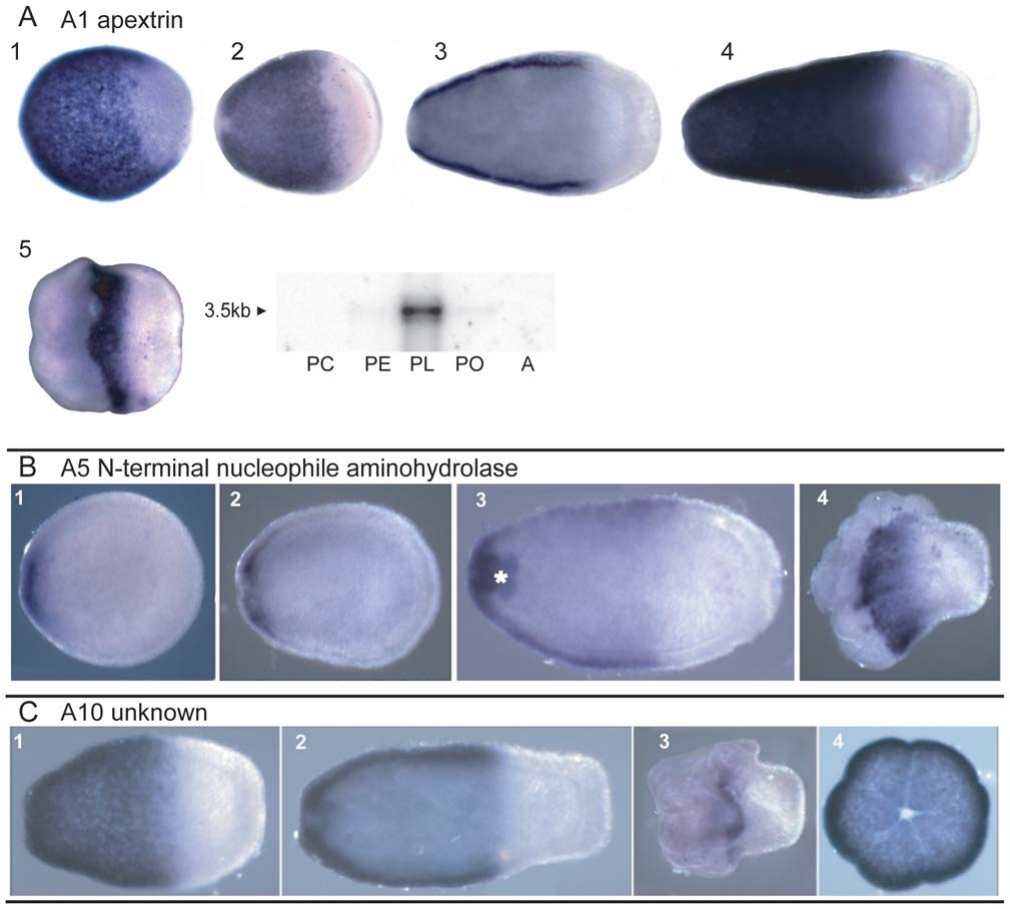
*In situ* hybridization - pool A transcripts. Three pool A transcripts show a similar pattern of ectodermal expression orally. (A) A1 (apextrin). Shortly after closure of the blastopore expression is in discrete patches with none at the aboral end (1). As it gradually elongates, expression continues in the oral two-thirds of the planula (2–4), finally ending as a belt separating the oral and aboral ends as the planula rounds up at the time of settlement (5). (B) A5 (N-terminal nucleophile aminohydrolase). Initial expression, shortly after blastopore closure, is limited to the oral ectoderm (1,2), but by the time the planula has elongated (3) expression is found in the oral two-thirds of the planula, similar to that observed for apextrin; the ectoderm of the developing siphonoglyph is also staining (white asterisk). Expression then resolves to a region in the middle as the planula begins to metamorphose (4). A10 (unknown function) shows a similar expression pattern. All pre-settlement stages are oriented with aboral to the right, as that end leads when swimming, while post-settlement stages are oral side up. Where available, the virtual northern blots are reproduced in this figure.

**Figure 5:**
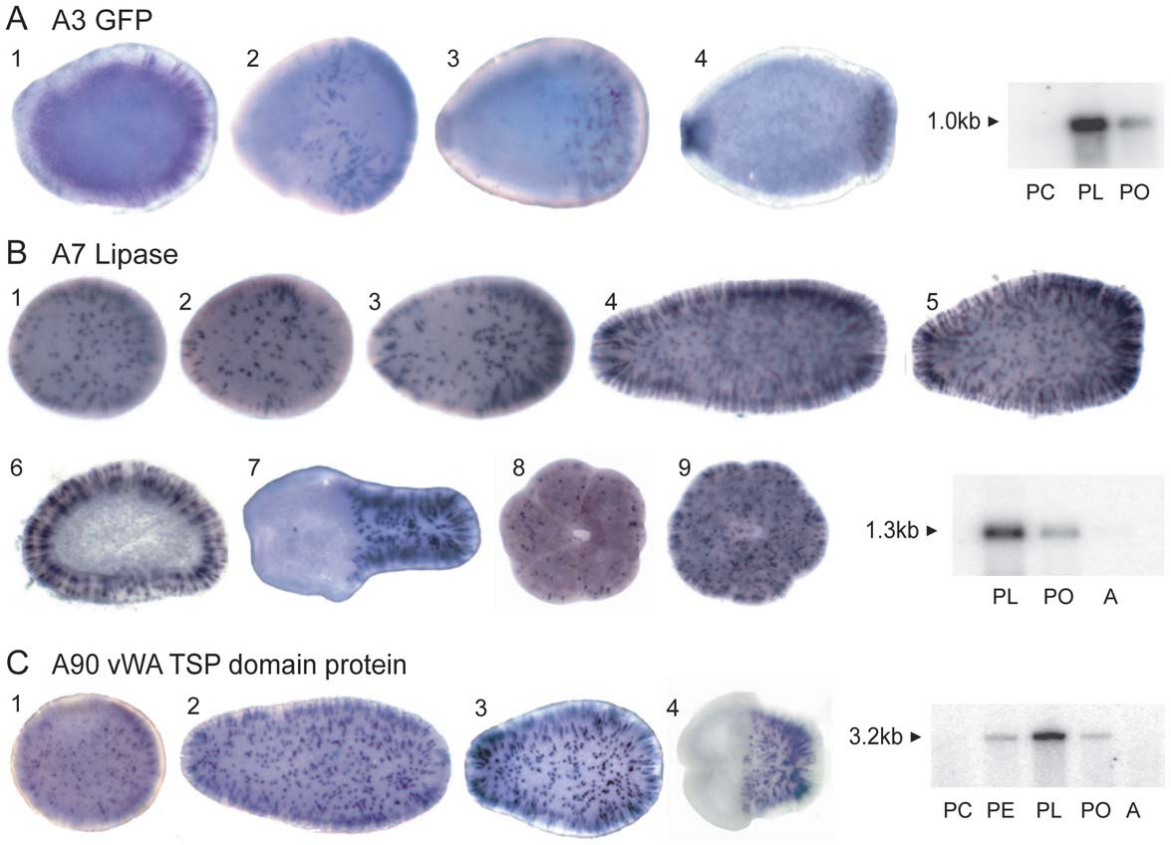
*In situ* hybridization - pool A transcripts. Three pool A transcripts are expressed pre-settlement in scattered ectodermal cells. (A) A3 (GFP). Early expression is in scattered ectodermal cells, with a biased distribution toward the aboral end of the developing planula (1–2). As development continues, expression appears surrounding the oral pore (3) and becomes more and more restricted to oral and aboral ends as development continues (4). (B) A7 (lipase) is expressed in abundant cells relatively evenly scattered in the ectoderm, from shortly after blastopore closure until settlement (1–6). At the time of settlement the oral part of some planulae ceases to express this transcript (7). In others, expression continues orally and aborally for a short time after settlement (8–9). 1–5 and 7–9 are whole mounts; 6 is a transverse section. (C) A90 (vWA TSP domain protein) has a similar expression pattern to that of A7. All pre-settlement stages are oriented with aboral to the right, as that end leads when swimming, while post-settlement stages are oral side up. Where available, the virtual northern blots are reproduced in this figure.

**Figure 6:**
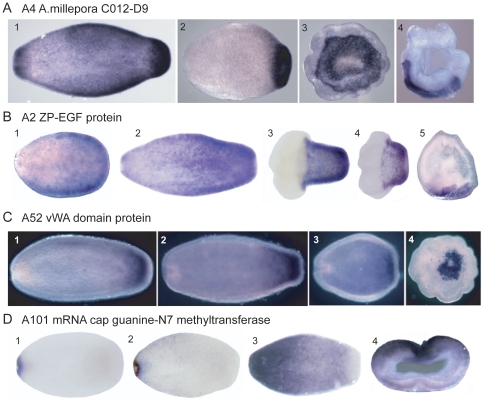
*In situ* hybridization - pool A transcripts. Pool A transcripts showing axially restricted expression patterns. (A) A4 (*A. millepora* C012-D9) is strongly expressed in the aboral two-thirds of the planula larva, with a band of minimal expression separating this expression from the strongly expressing oral end (1). Expression then becomes limited to the aboral end of the settling planula (2) and to the area of the future calicoblast layer (3–4). (B) A2 (EGF) Expression starts aborally in the pear stage (1) and remains strongest there throughout the planula and early settlement stages (2–4), becoming restricted to the future calicoblast layer at the time of settlement (5). (C) A52 (vWA domain protein). Expression is strongest in the aboral endoderm throughout the pre-settlement period (1–3) and continues in a restricted area at the aboral end post-settlement (4). (D) A101 (methyltransferase). In contrast to the other transcripts included in this figure, A101 is expressed orally throughout the stages investigated. Initially, in the early planula, it is expressed in a tight ring around the oral pore (1,2), while later expression forms a gradient running the full length of planula with the exception of the aboral end (3). Oral expression continues post-settlement (4). All pre-settlement stages are oriented with aboral to the right, as that end leads when swimming. Post-settlement stages A3 and C4 are shown looking directly onto the aboral surface, while A4 and B5 are oriented aboral side down.

**Figure 7:**
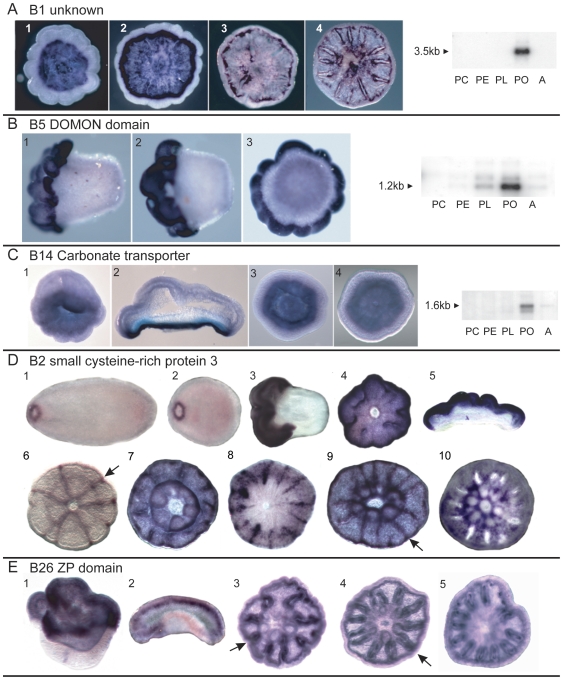
*In situ* hybridization- pool B transcripts. Genes up-regulated post-settlement. (A) B1 (unknown function) is sharply up-regulated at settlement and is expressed in aboral ectodermal cells in the future calicoblast layer except at the rim of the base. (1, 2). Expression then fades centrally, leaving a circle of expressing cells just inside the rim (3). Slightly later this circle of expressing cells is maintained and expression is also detected along the developing septa. (B) B5 (DOMON domain protein) is initially expressed orally at the time of settlement (1, 2), with post-settlement expression limited to the rim, in a pattern almost complementary to B1. (C) B14 (carbonate transporter) is expressed immediately post-settlement in the aboral ectoderm of the future calicoblast layer (1–4). (D) B2 (a SCRiP) is expressed in the planula as a ring around the oral pore (1, 2). At the time of settlement it becomes delimited to the oral ectoderm (3–5). Post-settlement it continues to be expressed orally in addition to being expressed along the septa (6–9, arrows in 6,9). In (10) the main expression is in a more central ring and between the tentacle bases of the central polyp. (E) B26 (ZP domain) is first expressed orally at settlement (1, 2) and later along the developing septa (3–5, arrows 3, 4). Where available, the virtual northern blots are reproduced in this figure.

**Figure 8:**
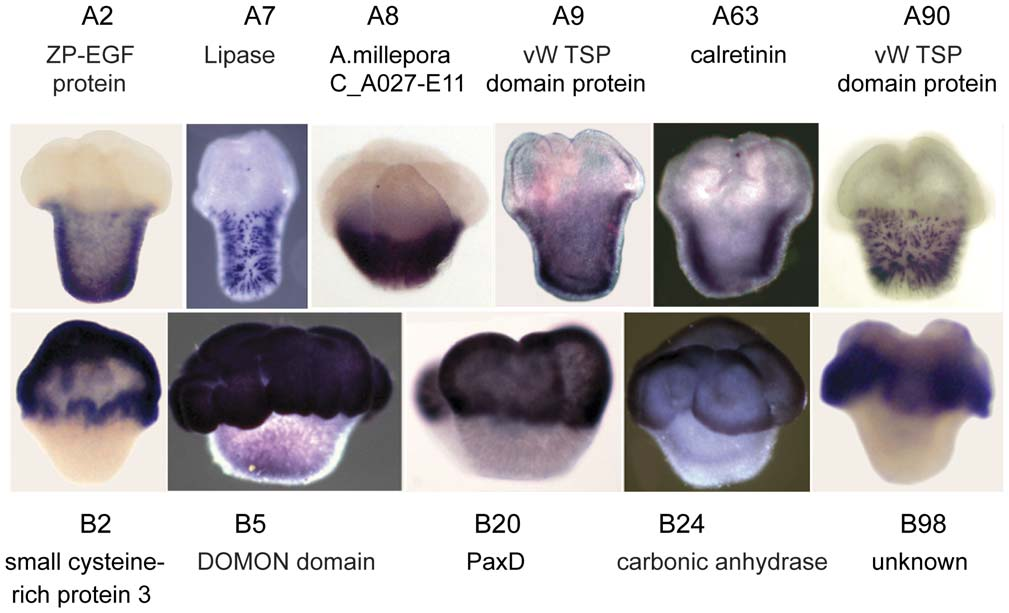
Examples of axially restricted expression patterns at settlement. Pool A transcripts expressed in the aboral region at settlement (top row). Pool B transcripts expressed in the oral region at the same stage (bottom row).

To investigate the spatial as well as temporal distribution of transcripts during development, selected sequences were used for whole mount in situ hybridization (Figures 3–8). In general the temporal expression of the clones seen with in situ hybridization is consistent with the pool from which they derive, i.e. clones corresponding to pool A sequences give a stronger signal in planula larvae than in later developmental stages, and conversely, clones corresponding to pool B sequences give a stronger signal in the post-settlement stages than in the planula. Many of the cDNAs show dynamic spatial, or tissue specific, as well as temporal patterns of expression.

Three of the cDNAs derived from the pool A sequences have strikingly similar patterns of expression (Figure 4A–C). In all three cases, expression is first observed in the early, pear-shaped planula, at the oral end. This expression is weak at first but rapidly becomes stronger as development proceeds and eventually extends about two thirds of the body length from the oral end. There is a sharp boundary where the expression domain ends, with the aboral portion of the larva remaining free of staining. As the larva undergoes metamorphosis, expression of these three transcripts starts to fade, becoming restricted to a narrow belt in the center of the oral-aboral axis, corresponding to the future lateral margin of the polyp. Subsequent expression then differs between these sequences. In the case of A1, expression rapidly fades and is not detected post-settlement. A10 expression becomes confined to a rim at the edge of the basal disc, while A5 expression becomes confined to the oral portion in later post-settlement polyps.

The A1 transcript codes for the previously described *Acropora* Apextrin-Am, a protein containing a membrane attack complex/perforin domain (MAC/PF) [24]. The transcript A5 codes for a 369 amino acid protein which contains a CBAH (conjugated bile acid hydrolase) domain, found in enzymes which cleave non-peptide carbon-nitrogen bonds. Database similarity searches reveal that the predicted protein has highest similarity to bacterial choloylglycine hydrolases, with no significant similarity to any protein encoded by the human, *Drosophila*, *Caenorhaditis elegans* or *Saccharomyces cerevisiae* genomes. Interestingly, searches against the *Nematostella vectensis* and *Hydra magnipapillata* databases also produce no significant match. In addition to the CBAH domain the protein has an N terminal signal sequence and a putative C terminal endoplasmic reticulum retention signal, KEEL [27]. This protein may therefore function in a coral-, or taxon-specific process, involving processing and secretion of proteins in a spatially restricted domain of the larval ectoderm. The protein encoded by the A10 transcript also shows similarity only to bacterial proteins, but as these are un-annotated and have no conserved protein domains, its function remains unknown.

Three other pool A transcripts show expression in individual ectodermal cells (Figure 5A–C). Transcript A7 encodes a 352 amino acid protein with similarity to mammalian pancreatic lipase. The *Acropora* egg is lipid-rich and during gastrulation much of the lipid is internalized, resulting in a planula larva that consists of a thin ectodermal layer surrounding a lipid-filled endoderm. This lipid is used by the actively swimming larva as an energy source [28]. The expression of the gene is first detected in early swimming planulae in a subset of ectodermal cells distributed along the full length of the body. This expression increases as the planula develops and elongates. During metamorphosis the expression in some specimens becomes restricted to the aboral region, while others continue oral expression for a short time after settlement.

Although showing a strikingly similar expression pattern, transcript A90 codes for an unrelated putative extracellular protein. Expression during metamorphosis becomes restricted to the aboral region. As metamorphosis proceeds, expression of A90 fades.

Transcript A3, coding for a larval fluorescent protein, also shows expression in discrete ectodermal cells. Extensive analysis of *Acropora* ESTs encoding fluorescent proteins indicates that there are red and green fluorescing types, which may represent paralogous sequences [25]. The overall similarity between these sequences is very high, and in situ hybridization cannot distinguish the two types. In the planula the expression intensifies at the two ends of the oral-aboral axis, which correspond to the areas that exhibit maximum fluorescence [25].

Other pool A transcripts also show developmentally regulated, axially restricted patterns of expression. Transcript A4 (Figure 6A), which was identified as *Acropora* C012-D9 mRNA [4] and encodes a putative secreted protein with no similarity to other proteins, transcript A2 (Figure 6B), encoding an EGF domain-containing protein, and transcript A52 (Figure 6C), encoding a putative extracellular protein containing a von Willebrand factor A domain, all show expression predominantly in the aboral region of the planula larva. During metamorphosis, expression of these three transcripts remains confined to the aboral ectoderm. Other pool A transcripts that show expression predominantly in the aboral region of the planula and metamorphosing larva are A9 (Figure 8), encoding an extracellular von Willebrand factor A domain-containing protein, A8 (*Acropora* GS01TE02 mRNA, identified in a microarray analysis of the response of coral larvae to inducers of settlement and metamorphosis (Figure 3 of [20])), encoding a protein of unknown function, and A63, encoding calretinin (Figure 8). In contrast, transcript A101 (Figure 6D), encoding a mRNA cap guanine-N7 methyltransferase, shows expression around the oral pore in the early planula. This expression then spreads to a wider area, still restricted to the oral half of the axis.

The expression patterns for three highly up-regulated pool B transcripts are shown in Figure 7A–C. Transcript B1 encodes a putative secreted protein which has no similarity to any sequence in the public databases. There is no expression in the planula, in accordance with the virtual northern blot (Figure 7A). Expression starts at the onset of metamorphosis in the aboral region. As metamorphosis continues, the expression intensifies and is confined to the ectoderm of the aboral surface. Later expression can be seen in the ectoderm associated with the developing septa. Transcript B5 is equivalent to *Acropora* C_A005-G11 mRNA [20] and encodes a protein containing a DOMON domain. This protein also contains a putative transmembrane domain at the carboxy terminus and is predicted to be a type I transmembrane protein, with the bulk of the protein outside the plasma membrane. DOMON domains have been implicated in heme and sugar recognition [22], and extracellular adhesion [29]. Expression of this transcript is confined to the oral half of the metamorphosing larva (Figure 7B, and [20]); the aboral region remains clear of expression. Transcript B14, encoding a HCO3 transporter, is expressed strongly in the aboral ectoderm after metamorphosis has commenced (Figure 7C). Transcript B2 encodes a small cysteine-rich protein, SCRiP3 [20], [30], a member of a family of coral-specific proteins of unknown function. Expression of this transcript is first detected as a ring surrounding the oral pore in the planula. As metamorphosis proceeds, the expression increases in intensity and area to cover the oral half of the larva. Later, expression is seen in tissue associated with the developing skeletal septa. Transcript B26 encodes a putative extracellular protein containing a Zona Pellucida (ZP) domain. ZP domains are found in a variety of extracellular matrix proteins, where they are involved in protein polymerization [31], [32]. Expression is seen on the oral surface of the settling larva, and as metamorphosis proceeds becomes localized to tissue associated with the developing septa (Figure 7E).

Other pool B transcripts that show a higher level of expression in the oral region include B20, encoding the transcription factor PaxD [5], [33], B24 (equivalent to *Acropora* A030-E11 mRNA, [4], [20]), encoding a carbonic anhydrase, and B98, encoding a putative secreted protein of unknown function (Figure 8). In addition, several pool B transcripts whose expression has been described previously are expressed at higher levels orally. Among these is B15 (equivalent to *Acropora* B041-G7 mRNA, [20]), which encodes a putative secreted protein of unknown function, but which has similarity to a sequence isolated during a screen for transcripts differentially expressed during ascidian metamorphosis [17]. It is expressed in the oral half of the settling larva and fades away after metamorphosis. B4 (equivalent to *Acropora* A036-E7 mRNA, [4]), encodes a protein with similarity to a haemolytic lectin CEL-III from the sea cucumber *Cucumaria echinata* [34]. It is expressed in the oral ectoderm of pre-settlement planula larvae and on the oral side of the newly settled polyp [4]. CEL-III may form ion-permeable pores after binding to cell surface carbohydrates, leading to rupture of the cell membrane [34]; possible functions of *Acropora* B4 are in the cellular remodeling accompanying metamorphosis or in defense against attacks by micro-organisms. Transcript B3 is equivalent to *Acropora* A047-G9 mRNA [20] and encodes a 334 amino acid protein with an N terminal signal peptide. This protein has no significant similarity with other sequences apart from some *Nematostella* proteins. During metamorphosis, expression is confined to the oral portion of the settling larva and persists orally after settlement. In contrast, transcript B49 (equivalent to *Acropora* A043-D8 mRNA, [4]), which encodes a C-type lectin, is expressed in scattered ectodermal cells that are more abundant aborally both pre- and post-settlement. This transcript is similar to a C-type lectin showing transcriptional repression during thermal stress in the coral *Pocillopora damicornis* [15].

Of the genes tested by in situ hybridization which showed an axially restricted pattern, many of the pool A transcripts (8 of 13) were expressed in the aboral region of the planula, settling polyp, or mature polyp. Conversely, many of the pool B transcripts (9 of 12) were expressed in the oral region. Figure 8 shows settling polyps expressing some of the axially restricted pool A and pool B transcripts. This confirms the pattern reported in our latest microarray study [20], although the significance of these patterns will remain unknown until we know more about the functions of the genes expressed in this way.

### Comparison to microarray

**Figure 9:**
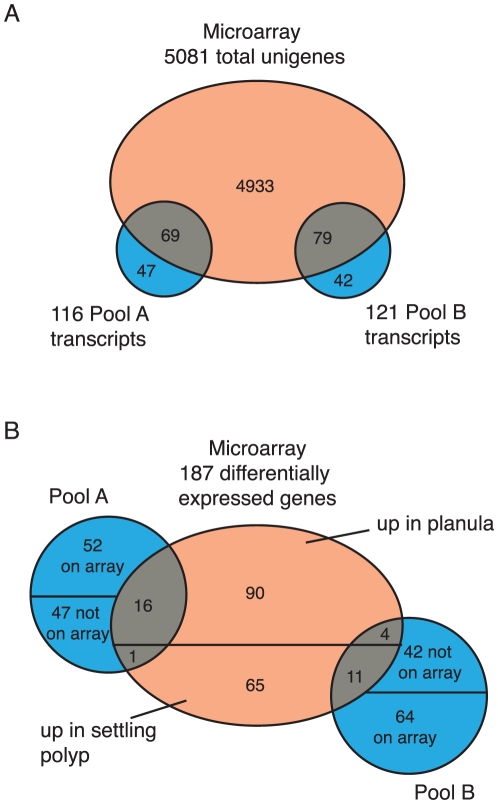
Comparison of SSH with microarray. A. Overlap between pool A and pool B transcripts (blue) and all microarray unigenes (orange). B. Overlap between pool A and pool B transcripts and differentially expressed microarray unigenes.

We have previously carried out a large scale study of transcriptional changes during *Acropora* development using cDNA microarrays [4], including a comparison of the pre-settlement planula with the newly settled polyp, as well as a more recent study focused specifically on a comparison before and after experimentally induced settlement [20]. The material used for the first microarray study was directly comparable to that reported on here, so only that comparison will be discussed below. Due to the similar starting material it might be expected that there would be a correlation with the present study, i.e. pool A transcripts should be found to correspond to unigenes that are expressed at higher levels in the planula according to the microarray and, conversely, pool B transcripts should be found to be expressed more highly in the polyp. Figure 9 and Table 3 summarize the results of this comparison. Sixty-nine of the pool A transcripts were also represented on the microarray, while 79 of the pool B transcripts were represented (Figure 9A). Pool A contained 17 of the 187 transcripts that were differentially expressed between the planula and newly settled polyp in the microarray experiment (expression clusters CII and CIV [4]; Figure 9B). Of these, 16 were found to be expressed at a higher level in the planula, while one was found to be expressed at a higher level in the settled polyp. In the case of the pool B transcripts, fifteen corresponded to differentially expressed unigenes according to the microarray study (Figure 9B). Eleven of these were expressed at a higher level after settlement. The remaining four were expressed at a higher level in the planula.

**Table 3:** Comparison of SSH with microarry.

Transcript	Name	Microarray fold change
A1	MACPF apextrin	+3.3
A3	red fluorescent protein	+2.2
A5	N-terminal nucleophile aminohydrolase	+4.2
A6	Pyridoxal-dependent decarboxylase	+2.3
A7	Lipase	+9.8
A8	unknown	+5.9
A9	vW TSP domain protein	+1.4
A10	unknown	+6.1
A37	zinc finger protein	+2.4
A52	vWA domain protein	+1.5
A65	poly A binding protein	+1.4
A74	FKBP-type peptidyl-prolyl cis-trans isomerase	+1.9
A86	unknown	+2.0
A90	hemicentin-1	+4.7
A93	Trefoil domain protein	+4.6
A101	mRNA cap guanine-N7 methyltransferase	−2.5
A102	vW TSP domain protein	+1.8
B1	unknown	−2.7
B2	small cysteine-rich protein	−6.2
B3	unknown	−6.7
B4	CEL-III Lectin	−1.9
B5	DOMON domain	−1.7
B6	Asp/Glu racemase	+1.7
B15	transmembrane receptor	−1.7
B24	carbonic anhydrase	+2.5
B43	transporter	−2
B49	C-type lectin	−3.2
B63	Pirin	−1.4
B72	unknown	+2.2
B98	unknown	−15.3
B99	SH3 and PX domains 2A	+1.4
B107	unknown	−4.1

Pool A and B transcripts which correspond to cDNAs showing significant (p<0.05) difference in expression levels according to the microarray. Gene expression fold changes are shown; where these are derived from multiple spots the median value is given. Positive values indicate higher expression in the planula relative to the post-settlement stage; negative values indicate higher expression in the post-settlement stage relative to the planula.

Of the remaining 99 pool A transcripts, 52 correspond to unigenes on the microarray, but failed to show significant differential expression there, and 47 were not represented on the microarray (Figure 9B). Two of the pool A transcripts tested by virtual northern, A61 (Figure 2) and A108, were not represented on the microarray but show a difference in expression level (Figure 2), while three (A2, A63 and A78) were represented on the array, but failed to show differential expression by that technique.

For pool B, 64 transcripts had counterparts on the microarray that did not show significant differential expression, while 42 transcripts were not represented (Figure 9B). As was the case for pool A, two virtual northerns (B14 and B34) show differential regulation of pool B transcripts that are not represented on the microarray, while two (B26 and B62) did not show significant differential expression on the microarray (Figure 2). In one case, B24, the virtual northern shows a slightly higher level of expression in the post-settlement stage, whereas the microarray indicated a higher level in the planula.

## Discussion

The suppressive subtractive hybridization (SSH) method of the PCR-Select system (Clontech) was used to produce two pools of cloned cDNA sequences corresponding to transcripts whose expression is higher in pre- or post- settlement stages of the coral, *Acropora millepora*. As well as enriching for differentially expressed sequences, the technique uses hybridization kinetics to reduce the relative concentration of highly abundant sequences while increasing that of rare transcripts. In spite of this, sequences corresponding to some highly abundant transcripts were recovered multiple times. Possibly, this is because the transcripts are expressed at levels too high for the normalization process to work completely. This explanation is consistent with the observed intensity of hybridization on virtual northern blots, and the frequency with which clones corresponding to some of the transcripts were recovered from cDNA libraries.

The subset of transcripts tested by virtual northern blot all showed higher expression in the appropriate stage, indicating that the subtractive hybridization was successful. In addition, the fact that none of the sequences was found in both pool A and pool B lends support to the notion that they represent differentially expressed transcripts, rather than transcripts which are equally abundant in both stages and which appear in the pools as a result of a failure of the PCR-Select technique.

The results presented here are broadly consistent with a previous cDNA microarray experiment. Of the 187 unigenes found to be differentially expressed in a comparison between planulae and newly settled polyps in the array experiment [4], 32 were also isolated by the SSH process. For most of these transcripts there is agreement between the two techniques. Many of the transcripts showing high levels of differential expression using virtual northen blots were also identified by the microarray. In these cases the fold change in expression levels measured by the microarray is less than that measured by virtual northern blot. This is consistent with published reports that microarrays tend to underestimate relative changes in expression when compared to quantitative RT-PCR or northern blots [35], [36]. For one pool A transcript and four pool B transcripts there is a discrepancy between the array and SSH results. One possible cause of such disagreement is that the samples used to prepare the cDNA that was probed in the microarray experiment were collected during a separate spawning event from those used to make the cDNA for the SSH study. Environmental differences between the two years may have affected gene expression, resulting in different transcripts being identified. Also, because a number of the transcripts identified by SSH show a sharp up or down-regulation between the two stages compared it is possible that slight differences in the ages of the specimens sampled could affect the relative expression levels. It should also be noted that Seneca et al [37], using qPCR, found large differences in the response to heat stress in A. millepora colonies growing near each other on the Magnetic Island reef flat, and Meyer et al [38], working with crosses from the same Magnetic Island population used in our study, found, also using qPCR “extensive variation in …responses depending on genetic background, including qualitative differences (i.e. up-regulation in one family and downregulation in another)”.

Almost half of the transcripts identified by SSH are represented on the microarray but fail to be classed as differentially regulated. Although the differential expression of most of these transcripts has not been verified, virtual northern blots for some confirm their differential expression in this study. These differences between techniques could also be due to the sample variability discussed above. Alternatively, the SSH methodology may be more sensitive, and the normalization procedure means that rare transcripts may be more likely to be identified.

Over one third of the transcripts identified by SSH were not represented on the microarray, reflecting the fact that the 5081 unigenes represent only approximately 25–30% of the transcriptome. On the other hand, the majority of unigenes found to be differentially expressed by the microarray were not recovered by the SSH. This could be because the present study is also far from exhaustive; since most of the identified transcripts were represented by a single SSH clone, it is likely that extending the study would result in the identification of more differentially expressed transcripts.

The expression of many of the identified transcripts was restricted along the oral-aboral axis. The expression patterns shown in Figure 5 resemble each other in that each of these genes is expressed in isolated ectodermal cells in the planula, with expression being down-regulated at settlement. In the case of green fluorescent proteins (GFPs), despite considerable speculation, experimental proof of function is lacking [26], [39]. This specific GFP is expressed in immature stages of *Acropora*, whereas others are specifically expressed in the adult [40]. A role in the photoprotection of zooxanthellae has been suggested in *Acropora* adults (e.g. [41], but see [42]), but *Acropora* planulae generally lack zooxanthellae [43]. Heat and light stress cause a small reduction in the fluorescence emanating from these cells and the brighter their fluorescence the less likely planulae are to settle [26]. While the exact role of the lipase gene (A7) remains unknown, it is likely to be involved in the breakdown of the endodermal storage lipids that are the primary energy source for the planktonic phase of coral development. The temporal expression of the gene is consistent with use of lipid stores during the energetically demanding period of pre-settlement motility and of the metamorphic process. In addition, the depletion of lipid reserves during the latter part of larval life may result in a decrease in buoyancy which may facilitate settlement. Finally, A90, a molecule coding for a protein with vWA and thrombospondin domains, could be involved in several processes related to cell movement and changes in cell adhesion associated with settlement and metamorphosis.

Three pool A transcripts forming a synexpression group were expressed in a domain comprising about two thirds of the axis from the oral end and having a sharp cutoff reminiscent of the *Drosophila* gap genes. Later, in the settling polyp, the expression of all three genes resolves into a band around the mid point of the oral-aboral axis. The function of these genes is unclear; although they may appear to be involved in regional specification of the body axis they do not code for transcription factors as do the gap genes in *Drosophila*. One of the genes is *Acropora* apextrin, a gene coding for an innate immunity protein containing a perforin domain [24] while the other two show similarity only to bacterial proteins.

Several other transcripts show a similarly abrupt axial restriction, particularly at the early stages of metamorphosis. At this stage, six of the pool A transcripts are expressed only in the aboral half of the axis, while five of the pool B transcripts are expressed exclusively in the oral region. Thus in a short space of time, the apparently uniform ectoderm of the planula larva gives rise to two regions with very different gene expression profiles, reflecting their future fates and functions; the aboral region will contact the substratum and form the calcifying tissue, the calicoblastic ectoderm responsible for laying down the skeletal structures of the polyp, while the oral region is in contact with the marine environment and will give rise to the column and feeding mouthparts of the polyp, which subsequently will bud and branch to give rise to a new colony. Overall, thirteen of the pool A transcripts showed an axially restricted expression pattern and of these, eight were predominantly expressed in the aboral region, and a further three (mentioned above) defined a band across the middle of the settling polyp. Ten of the pool B transcripts had an axially restricted pattern of expression and eight of these were predominantly expressed orally. This idea of temporal waves of axially restricted gene expression is in agreement with gene expression changes seen in response to inducers of settlement in *Acropora* [20].

Although the numbers of transcripts identified in this study are small, there are some striking differences in the composition of certain classes of differentially-regulated genes between those up-regulated pre- and post-settlement. For example, in the category “extracellular matrix/cell adhesion” four genes coding for vWA domain proteins are up-regulated pre-settlement. While vWA-containing proteins are involved in diverse processes, it is possibly significant that expression of two of these genes is in the aboral ectoderm, which will later give rise to the calicoblast layer that will be involved in secretion of the aragonite skeleton. There is a precedent for involvement of vWA domain-containing proteins in calcification in pearl oysters belonging to the genus *Pinctada*, where the PIF 97 proteins in *P. furcata* [44] and *P. margaritifera* [45] play key roles in the deposition of the nacreous layer. In contrast, post-settlement, three DOMON domain-containing protein genes are up-regulated, while none was up-regulated pre-settlement. These domains typically function in protein-protein interactions [29] and in the present case may function specifically in heme or sugar recognition [22]. Additionally, two genes coding for Ets domains are up-regulated post-settlement, with none up-regulated pre-settlement. Genes containing this domain play roles in the control of calcification in vertebrates [46] and sea urchins [47]. It will be interesting to see whether these differences in the subclasses of genes expressed pre- and post-settlement hold up in further investigations of differential gene expression.

Seventeen (fifteen percent) of the pool A transcripts and twenty-six (twenty percent) of the pool B transcripts did not have significant similarity to any sequence in the public databases. In some cases this is likely to be due to the incomplete nature of the transcriptome database, such that only a truncated, perhaps non-coding, region of the predicted transcript is available. In other cases, however, where the predicted sequence contains a long open reading frame, or the sequence has been independently confirmed by sequencing of cDNA clones, these sequences are likely to represent novel, or coral-specific proteins. Such taxonomically restricted genes (TRGs) are candidates for involvement in taxon-specific processes [48].

One of our goals in carrying out this screen was to discover genes potentially involved in calcification, which begins at settlement in corals. Several of the genes uncovered here are potentially involved in this process based on either their putative function or their expression. An obvious candidate on both counts is B14, a carbonate transporter, which is expressed immediately post settlement in the calicoblast layer as it is forming (Figure 7C). Others include B1, which is strongly up-regulated at settlement and is initially expressed in cells adjacent to the forming basal plate (Figure 7A1–3) and later along the forming septa (Figure 7A4). Another gene of interest in this context is B2 (Figure 7D), which, although it is initially expressed orally (Figure 7D1–5), later comes to be expressed along the septa (Figure 7D6–10). There are also genes with peak expression pre-settlement which, based on their expression, could be involved in preparations for calcification in the cells of the aboral end. These include A4 (a TRG; Figure 6A), A2 (EGF domain; Figure 6B), and A52 (vWA domain; Figure 6C), all of which are expressed in the aboral ectoderm at the time of settlement. Additional genes with aboral expression at the time of settlement include A63 (calretinin; Figure 8), A9 and A90 (both vWA genes; Figure 8) and A8 (a TRG; Figure 8). In addition, B85, coding for galaxin-like2, a putative organic matrix protein, was identified in this SSH study as up-regulated post-settlement, consistent with a previously published virtual northern blot [49].

Of the 237 transcripts described here, 89 were not represented in a previous microarray study [4], which highlights the fact that many *Acropora* genes are still to be discovered. To the extent that they were checked with virtual northern blots both the microarray and SSH studies appear to be internally consistent, so only further studies will confirm which genes are consistently differentially expressed as a result of developmental changes, as opposed to other environmental factors which may also influence gene expression.

New technologies are rapidly changing the way in which alterations in gene expression can be assayed. High throughput transcriptome sequencing is becoming an accessible technique enabling the relative abundances of transcripts to be measured, as well as providing a more complete coverage of the transcriptome than can be obtained by cDNA microarrays or techniques such as the present study. The use of qPCR in place of virtual northern blots would enable more precise estimates of changes of levels of gene expression; however virtual northern blots are a useful, low cost method for comparing expression levels of multiple sequences, while in addition providing information about transcript size and possible splice variants. In addition, there are presently no generally accepted internal control genes for qPCR in corals; recent studies on A. millepora have used non-overlapping sets of three genes [37] [38].

The present study using SSH and Sanger sequencing resulted in a relatively small number of differentially expressed genes compared to either microarray or high throughput sequencing. However, rather than stopping with a list of genes, we have then used a combination of sequence analysis, literature search and spatial expression data obtained using in situ hybridization in an attempt to understand the roles of these genes in greater detail. The clues obtained by these methods will remain our best indicators of coral gene function until such time as genetic tools like those presently available for *Drosophila* and *Caenorhabiditis* become available for corals, and in the short term constitute our best hope for understanding how genes control coral development.

## Materials and Methods

### Biological material

Staged embryos and larvae were collected during the annual mass spawning event at Magnetic Island, Queensland, Australia under permit G08/28473.1 issued to Prof. David Miller by the Great Barrier Reef Marine Park Authority. Specimens for RNA isolation were frozen in liquid nitrogen and subsequently stored at −80°C. Specimens for in situ hybridization were fixed as previously described [50].

### RNA isolation

Total RNA was isolated from frozen material ground in liquid nitrogen using the RNAwiz reagent (Ambion) according to manufacturer's instructions. Poly A+ RNA was prepared using the polyATtract system (Promega).

### Subtractive hybridization

cDNA was prepared from settlement-competent planula larvae (pre-settlement) and newly metamorphosed primary polyps (post-settlement) using the Clontech PCR-Select cDNA Subtraction Kit. Two reciprocal subtractive hybridizations were carried out according to the manufacturer's instructions: one using the pre-settlement cDNA as driver and the other using the post-settlement cDNA as driver. The subtracted PCR products were ligated into pGEMT easy (Promega) and transformed into *E. coli* DH5alpha cells, resulting in two pools of clones enriched for sequences preferentially expressed in either the pre- or post-settlement stage. Plasmid DNA was prepared from single colonies and sequenced using vector primers.

### Sequencing and annotation

DNA sequencing was carried out using DyenamicET terminators (Amersham) or Big Dye Terminator v. 3.1 (Applied BioSystems). Reactions were run on ABI377 or ABI 3730 sequencers at the Biomolecular Resource Facility (JCSMR, ANU). Sequences were analysed using MacVector 7.2.2. (Accelrys) and Lasergene (DNASTAR). Sequences were assigned to functional categories based on the results of blast and protein domain searches (rpsblast). Gene Ontology annotations were obtained using Blast2GO (http://www.blast2go.org/) [51]. Data for the microarray study [4], to which the present study is compared, have been deposited in the Gene Expression Omnibus (GEO) database (accession GSE11251). Sequences reported here are available from GenBank (accessions JK342251–JK342530 and JN631071–JN631101).

### Virtual Northern Blotting

cDNA made from mRNA from staged embryos and larvae was subjected to controlled PCR amplification using the Clontech SMART™ PCR cDNA Synthesis Kit. After gel electrophoresis, the cDNA was blotted onto HybondN (Amersham). Probes were amplified from plasmids using the Nested PCR primer 1 and Nested PCR primer 2R (Clontech) and labeled with 32P using the Prime a Gene Labeling system (Promega). Probe sequences are given in Supporting Information Table S5. Hybridization and washes were carried out using standard techniques [52]. Filters were exposed on a phosphor screen which was scanned in a Molecular Dynamics PhosphoImager, and images were processed with ImageQuant v1.2 to enable measurement of relative expression levels.

### Isolation of full length sequences

cDNA libraries constructed in Lambda ZAP (Stratagene) were made from pre- and post-settlement material according to the manufacturer's instructions. The libraries were screened using standard techniques with 32P labeled probes made from selected sequences. Positive clones were recovered as inserts in pBluescript SK- and sequenced using vector and sequence-specific primers.

### *In situ* hybridization

Probe preparation, hybridizations and washes were carried out as previously described [50]. Clearing and photography were as described in [33]. Micrographs have been adjusted in Adobe Photoshop to make expression patterns as clear as possible. Where dark backgrounds hindered viewing of the staining pattern the background was removed using Photoshop, leaving the embryos on a white background.

## Supporting Information

Figure S1 **Frequency of isolation of SSH sequences.** The number of times sequences were independently isolated from the pools of clones is plotted against the number of sequences. Most sequences were isolated only once.
(EPS)

Table S1 **Pool A transcripts.** The columns contain the following information: column A, the transcript number as referred to in the text: * indicates that the transcript has been verified by cDNA clone sequencing; otherwise the transcript is predicted from the transcriptome database; column B, the length of the transcript; column C, the functional class of the protein for which the gene codes; column D, transcript name; column E, the number of independently isolated SSH clones corresponding to each transcript; column F, the number of separate pool A sequences corresponding to each transcript; column G, accession of the best hit resulting from a blastp search of the NCBI nr database. Hits with an E value of >1E-5 were considered non-significant; column H, description of the blast hit; column I, the E value of the best blast hit; column J, the sequence of the predicted protein; and column K, the transcript sequence.
(XLS)

Table S2 **Pool B transcripts.** The columns contain the following information: column A, the transcript number as referred to in the text: * indicates that the transcript has been verified by cDNA clone sequencing; otherwise the transcript is predicted from the transcriptome database; column B, the length of the transcript; column C, the functional class of the protein for which the gene codes; column D, transcript name; column E, the number of independently isolated SSH clones corresponding to each transcript; column F, the number of separate pool A sequences corresponding to each transcript; column G, accession of the best hit resulting from a blastp search of the NCBI nr database. Hits with an E value of >1E-5 were considered non-significant; column H, description of the blast hit; column I, the E value of the best blast hit; column J, the sequence of the predicted protein; and column K, the transcript sequence.
(XLS)

Table S3 **Pool A GO categories.** The columns contain the following information: column A, the transcript number as referred to in the text; column B, the hit-description; column C, the GO group, column D, the GO identification, column E, the GO term.
(XLS)

Table S4 **Pool B GO categories.** The columns contain the following information: column A, the transcript number as referred to in the text; column B, the hit-description; column C, the GO group, column D, the GO identification, column E, the GO term.
(XLS)

Table S5 **Probe sequences.** The columns contain the following information: column A, the transcript number; column B, the nucleic acid sequence of the probes used for virtual northerns; column C, the nucleic acid sequences of the probes used for in situ hybridization.
(XLS)
